# Serum levels of total IgE and soluble CD23 in bronchial asthma

**DOI:** 10.1155/S0962935196000075

**Published:** 1996-02

**Authors:** G. Di Lorenzo, P. Mansueto, M. Melluso, G. Morici, D. Cigna, G. Candore, C. Caruso

**Affiliations:** 1 Istituto di Medicina Interna e Geriatria Università di Palermo via del Vespro 141 Palermo 90127 Italy; 2 Istituto di Patologia Generale Università di Palermo Corse Tukory 211 Palermo 90134 Italy

## Abstract

The aim of the present study was to compare, during the pollen season, serum levels of total IgE and soluble CD23 (sCD23) from patients with allergic bronchial asthma, with those from healthy subjects. Significantly higher levels of total IgE and sCD23 were found in patients with asthma compared to the control group. Both in normal controls and in asthmatic patients, a significant correlation was shown between the levels of these two molecules. In asthmatic patients, significant correlations were found for both total IgE and sCD23, with lung function measured as bronchial responsiveness to inhaled methacholine. These results suggest that in asthmatic patients, in addition to the study of total serum IgE levels, the assessment of sCD23 serum levels may be helpful in the evaluation of disease activity.


Mediators of Inflammation 5, 43–46 (1996)

### Serum levels of total IgE and soluble CD23 in bronchial asthma

G. Di Lorenzo,[1] P. Mansueto,[1] M. Melluso,[1] G. Morici,[1] D. Cigna,[2] G. Candore[2] and C. Caruso[2,CA]

[1]Istituto di Medicina Interna e Geriatria, Università di Palermo, via del Vespro 141, 90127 Palermo, Italy; [2]Istituto di Patologia Generale, Università di Palermo, Corso Tukory 211, 90134 Palermo, Italy. Fax: (+39) 91 6555901.

[CA]Corresponding Author

**The aim of the present study was to compare, during the pollen season, serum levels of total IgE and soluble CD23 (sCD23) from patients with allergic bronchial asthma, with those from healthy subjects. Significantly higher levels of total IgE and sCD23 were found in patients with asthma compared to the control group. Both in normal controls and in asthmatic patients, a significant correlation was shown between the levels of these two molecules. In asthmatic patients, significant correlations were found for both total IgE and sCD23, with lung function measured as bronchial responsiveness to inhaled methacholine. These results suggest that in asthmatic patients, in addition to the study of total serum IgE levels, the assessment of sCD23 serum levels may be helpful in the evaluation of disease activity.**

**Key words:** Bronchial asthma, Pollen, Serum CD23, Serum IgE

### Introduction

It is well known that serum IgE levels are elevated in patients who suffer from atopic diseases.[1,2] Furthermore, in atopic asthma, IgE levels are recognized as a major risk factor for a variety of respiratory symptoms.[3] From both clinical and experimental evidence, asthma is now viewed as an inflammatory airway disease involving lymphocyte activation and the release of proinflammatory cytokines.[4–7] T-cells control IgE production by B-cells and activate nonspecific effector cells.[4,6–8] In atopic individuals, stimulation by allergens determines an increased synthesis of IgE and the up-regulation of CD23, i.e. the low affinity receptor for IgE (FcεRII), on a variety of cell types, and its release as a soluble molecule (sCD23).[9–12] The FcεRII/CD23 is expressed not only on B-lymphocytes but also on T-lymphocytes, monocytes, platelets and eosinophils, which may play a role in triggering IgE-mediated effector function.[9–12] This molecule, which is a member of the C-type animal lectin family, has been suggested to play a role in the regulation of IgE synthesis.[9–12,13] As mentioned previously, this membrane molecule may be transformed into a soluble form by limited proteolysis, i.e. shedding, as well as the cytokine and growth factor receptors and can then be detected in biological fluids, including serum.[14] Previous studies in adults with hyper-IgE syndrome have shown elevated serum levels of sCD23.[15] On the other hand, sCD23 has been suggested to be potentially useful in the diagnosis of atopic disease.[16]

A number of investigations have considered the association between atopic asthma and nonspecific bronchial responsiveness. The majority of those carried out, especially those employing histamine and methacholine tests to measure nonspecific bronchial responsiveness, have shown a significant association, when atopy was defined by an upper serum total IgE level.[17–19]

In this report, to gain insight into the significance of serum sCD23 in asthmatic patients, we have studied the serum levels of total IgE and sCD23 and the relationship between these variables in asthmatic patients with regard to their clinical status measured as bronchial responsiveness to inhaled methacholine.

### Patients and Methods

*Sample population:* Thirty-five subjects were studied; 25 were patients with bronchial asthma (15 women and ten men, range 19–54 years). All patients had *Parietaria* sensitization and a baseline forced expiratory volume in 1 s ($FEV_1$) of at least 80% of predicted value, a provocative concentration of inhaled methacholine causing a 20% fall in $FEV_1$ ($PC_{20}$) $< 4000$ μg/ml. All patients studied used inhaled bronchodilatators when needed and had not been treated with disodium cromoglycate in the preceding 7 days. None of the patients was using oral or inhaled corticosteroids. Inhaled bronchodilator therapy was withheld for 12 h before the study. The controls consisted of ten healthy women (range 18–40 years). None of these subjects had a history

© 1996 Rapid Science Publishers of prolonged disease and none was ill or taking any drug at the time of the study. The patients and the control group were studied during the *Parietaria* pollen season.

*Quantitation of serum total IgE:* IgE levels in sera were quantitated by Phadebas IgE PRIST® (Pharmacia, Uppsala, Sweden). Anti-IgE antibody, covalently coupled to a paper disc, was allowed to react with the IgE in standards and sera during the first incubation. After washing, $^{125}I$-labelled anti-IgE antibody was added. After washing, the radioactivity was measured by using a gamma counter. The amount of IgE was determined from the standard curve and expressed as UI/ml.

*Soluble CD23 assay:* Serum sCD23 levels were measured by a sandwich enzyme-linked immunoassay (Cellfree CD23 Test-Kit-Cell Sciences, Cambridge, MA, USA). Standards and samples were performed in duplicate. Absorbance was measured at 492 nm. The test was performed according to the manufacturer's instructions.

*Lung function measurements:* $FEV_1$ was measured with a Gould 2400 (Gould, Holland) automated system, taking the highest of three successive measurements, provided the difference between measurements was within 100 ml. A methacholine challenge was performed according to the method of Chai *et al.*[20] Increased concentrations were administered with a Mefar (Markos, Monza, Italy) nebulizer. After baseline measurements of $FEV_1$, subjects inhaled five puffs of saline, since that was considered as the control. Subjects then inhaled increasing concentrations of methacholine, ranging from 16 to 1 024 μg/ml. $FEV_1$ was measured 90 s after each concentration step. The provocation was terminated when $FEV_1$ fell by at least 20% from the post-saline value.

*Statistical analysis:* All data were expressed as means ± S.D. Correlations were calculated by linear regression. The values for the different groups were compared with Student's test.

### Results

Patients with allergic asthma had significantly higher serum levels of total IgE and of sCD23 than did the normal controls (Table 1). Both in normal controls (Fig. 1a) and in asthmatic patients (Fig. 1b), a significant correlation was shown between the levels of these two molecules.

To assess the role played by total IgE and sCD23 in the pathogenesis of airway obstruction, the blood concentrations of these products were correlated with changes in the objective measurements of bronchial hyperreactivity. Figs 2a and 2b, respectively, demonstrate that bronchial responsiveness (measured as the response to inhaled methacholine) significantly correlated with total IgE and sCD23 serum levels.

**Table 1.** Serum levels (mean ± S.D.) of total IgE and soluble CD23 in ten normal controls and 25 asthmatic patients

| | Healthy subjects | Patients |
|---|---|---|
| Total IgE | $87.0 \pm 19.4^a$ | $190.0 \pm 45.8^b$ |
| sCD23 | $84.4 \pm 29.7^c$ | $423.6 \pm 150.8^d$ |

a *vs.* b; c *vs* d.
$p < 0.001$.

### Discussion

Allergic processes are complex disorders in which inflammatory and immunological mechanisms are involved.[4] CD23 is an activation marker expressed on B-cells as they undergo isotype switching, and it acts as a low-affinity IgE receptor. The role of the FcεRII may well relate to the cell type on which it is expressed. Accordingly, the FcεRII on monocytes, platelets and eosinophils mediated IgE-dependent cytotoxicity and/or promotes phagocytosis of IgE-coated particles. Concerning B-cells, the newest function proposed is enhancement of antigen processing by nonspecific uptake of antigen IgE complexes by B-cells, that culminates in the highly efficient presentation of antigen fragments to histocompatibile, cognate T-cells. However, modulation of CD23 expression by cytokines and correlation of that modulation with IgE synthesis has been described. Accordingly, early studies demonstrated that elevated IgE levels were associated with elevated CD23 levels on B-cells. Although it is now clear that interleukin-4 (IL-4) was also involved in this up-regulation, other *in vitro* studies demonstrated that IgE alone was sufficient for CD23 up-regulation on lymphocytes, monocytes and eosinophils by protecting CD23 from degradation into sCD23. Thus interest in CD23 upregulation has centred largely on IL-4, primarily because this cytokine directly causes both IgE production and CD23 upregulation by increasing CD23 synthesis rates.[8–12,21]

CD23 is a labile protein in that a soluble fragment (sCD23) is released from cells. Previous work has found sCD23 levels in patients suffering from rhinitis, asthma and nonallergic asthma.[22] Studies in humans have found that sCD23 potentiates IgE synthesis although the manner is not known. Furthermore it has been

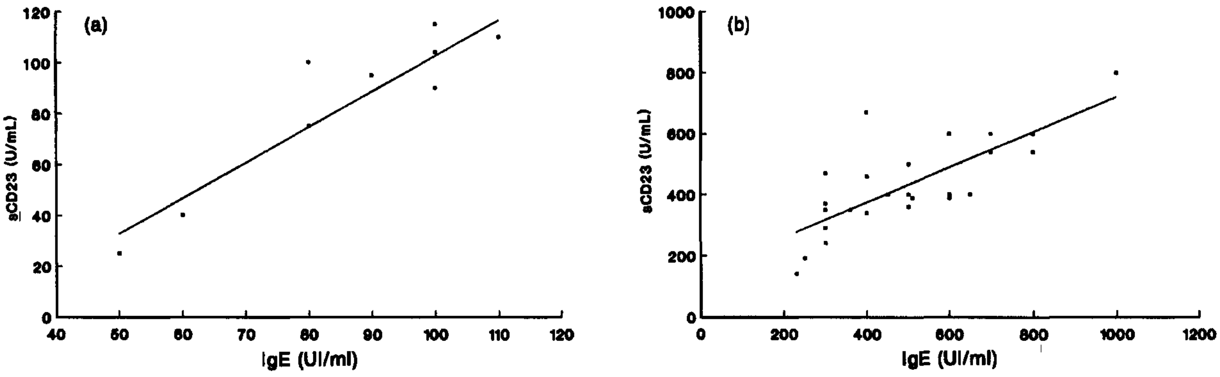

FIG. 1. (a) Correlation between total serum IgE values (UI/ml) and serum levels of sCD23 (U/ml) in ten normal controls ($r=0.91$; $p=0.0002$). (b) Correlation between total serum IgE values (UI/ml) and serum levels of sCD23 (U/ml) in 25 asthmatic patients ($r=0.76$; $p<0.0001$).

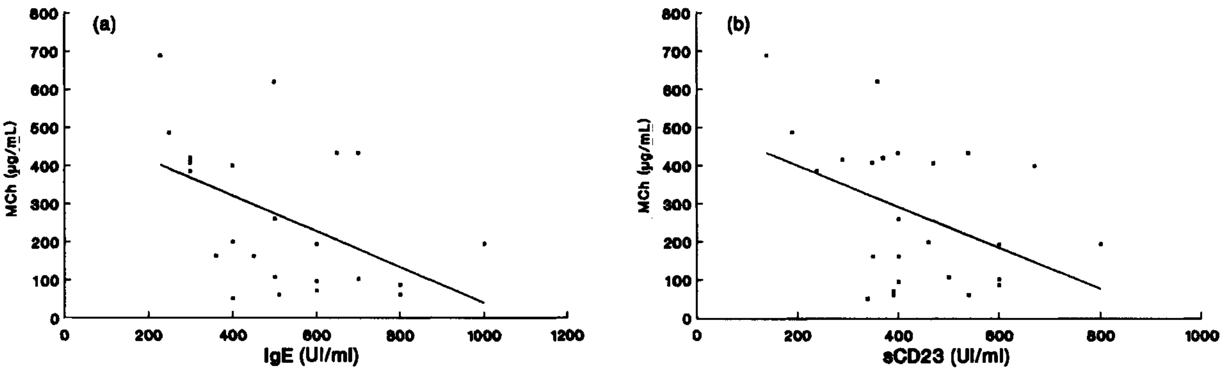

FIG. 2. (a) Correlation between total IgE values (UI/ml) and inhaled concentration of methacholine (MCh) (µg/ml) that determined the $FEV_1$ fell by at least 20% from post-saline value (see Patients and Methods) in 25 asthmatic patients ($r=-0.49$; $p<0.0001$). (b) Correlation between sCD23 values (U/ml) and inhaled concentration of methacholine (MCh) (µg/ml) that determined the $FEV_1$ fell at least 20% from post-saline value (see Patients and Methods) in 25 asthmatic patients ($r=-0.43$; $p=0.0001$).

claimed that sCD23 works as an autocrine B-cell growth factor and it plays a role as a macrophage inhibitory factor on monocytes. Soluble CD23 can now be acknowledged as an important pleiotropic cytokine. In fact, it has been recently shown to promote the differentiation of germinal centre B-cells, early thymocytes and early myeloid precursor cells. Besides culturing small, resting lymphocytes with soluble CD23, the molecule induces the synthesis of IgE *in vitro* when T-cells are present. In particular, IL-4 increases the precursor frequency of IgE-producing B-cells, whereas sCD23 induces IgE-committed B-cells to secrete relatively larger amounts of IgE.[8–12,21]

In our work, differences in sCD23 levels have been found in patients suffering from allergic asthma during the pollen season and in non-atopic patients. The serum total IgE levels were clearly higher in atopic patients, as classically established.[1,2] Our data show then that both in normal controls and in asthmatic patients there is a significant correlation between the levels of IgE and sCD23. As discussed above, this close correlation is likely mediated by IL-4 since high levels of IgE *per se* do not increase serum levels. Since previous studies have demonstrated a significant association between allergic asthma and non-specific bronchial responsiveness when atopy was defined by serum total IgE,[17–19] we have investigated the relationship between sCD23, total IgE and bronchial responsiveness. The results demonstrate that the blood concentrations of both products negatively correlated with changes in the objective measurements of bronchial hyperreactivity, measured as response to inhaled methacholine, confirming and extending previous reports.[19]

Asthma is a chronic inflammatory disorder of the airways, in which lymphocyte activation and the release of proinflammatory cytokines play a role. Bronchial hyperresponsiveness is a prominent feature in asthma.[4–7] Evaluation of immune activation is of potential value in monitoring patients with bronchial asthma. Our results suggest that in patients with asthma sCD23 may be helpful in the assessment of disease activity.

Future studies should determine the usefulness of monitoring sCD23 levels in individual patients with bronchial asthma for the prediction of the bronchial hyperresponsiveness and imminent broncho-obstruction.

### References

1. Lee B. IgE response and its regulation in allergic diseases. *Pediatr Clin N Am* 1988; **35:** 953–967.
2. Omenaas E, Bakke P, Elsayed S, Hanoa R, Gulsvik A. Total and specific serum IgE levels in adults: relationship to sex, age and environmental factors. *Clin Exp Allergy* 1994; **24:** 530–539.
3. Dolovich J, Zimmerman B, Hargreave FE. Allergy in asthma. In: Clark TJH, Godfrey S, eds. *Asthma*, 2nd ed. London: Chapman & Hall, 1983; 132–159.
4. Corrigan CJ, Kay AB. CD4 lymphocyte activation in acute severe asthma. Relationship to disease severity and atopic status. *Am Rev Respir Dis* 1990; **141:** 970–977.
5. Monteseirin J, Guardia P, Delgrado J, *et al.* Peripheral blood T lymphocytes in season bronchial asthma. *Allergy* 1993; **50:** 152–156.
6. Vidhcow JC, Olehling A, Boer L, *et al.* Pulmonary function, activated T cells, peripheral blood eosinophilia, and serum activity for eosinophil survival *in vitro*. A longitudinal study in bronchial asthma. *J Allergy Clin Immunol* 1994; **94:** 240–249.
7. Di Lorenzo G, Mansueto P, Melluso M, *et al.* Serum levels of soluble IL-2R, CD4 and CD8 in bronchial asthma. *Mediators of Inflammation* 1995; **4:** 270–272.
8. Walker C, Bode E, Boer L, *et al.* T cell subsets and their soluble products regulate eosinophilia in allergic and nonallergic asthma. *J Immunol* 1991; **146:** 1829–1835.
9. Capron A, Dessaint JP, Capron M, *et al.* From parasites to allergy: a second receptor for IgE. *Immunol Today* 1986; **7:** 15–18.
10. Gordon J, Flores-Romo L, Cairns JA, *et al.* CD23: a multi-functional receptor/lymphokine? *Immunol Today* 1989; **10:** 153–157.
11. Conrad DH. The Fc-RII/CD23: the low affinity receptor for IgE. *Annu Rev Immunol* 1990; **8:** 623–645.
12. Gordon J (ed). CD23. A novel multifunctional regulator of the immune system that binds IgE. *Monog Allergy* 1991; **29:** 1–208.
13. Romagnani S, Ricci M. Present views on the regulation of human IgE synthesis. *Immunol Today* 1990; **11:** 192–196.
14. Caruso C, Candore G, Cigna D, *et al.* Biological significance of soluble IL-2 receptor. *Mediators of Inflammation* 1993; **2:** 3–21.
15. Yanagihara Y, Sarfati M, Marsh D, *et al.* Serum levels of IgE-binding factor soluble (CD23) in diseases associated with elevated IgE. *Clin Exp Allergy* 1990; **20:** 395–401.
16. Kim KM, Inoue Y, Uenoyama Y, *et al.* Prediction of the development of atopic symptoms in early childhood by cord IgE-binding factors (soluble FcεRII). *Immunol Lett* 1990; **24:** 63–68.
17. Grainger DN, Stenton SC, Avery AJ, *et al.* The relationship between atopy and non-specific bronchial responsiveness. *Clin Exp Allergy* 1990; **20:** 181–187.
18. Burney PGJ, Britton JR, Chinn S, *et al.* Descriptive epidemiology of bronchial reactivity in adult population: results from a community survey. *Thorax* 1987; **42:** 38–44.
19. Cookson WOCM, Musk AW, Ryan G. Association between asthma history, atopy, and non-specific bronchial responsiveness in young adults. *Clin Allergy* 1986; **16:** 425–432.
20. Chai H, Farr RS, Froehiic IA, *et al.* Standardization of bronchial inhalation challenge procedures. *J Allergy Clin Immunol* 1975; **56:** 323–327.
21. Mudde GC, Bheekha R, Bruijnzeel-Koomen CAFM. Consequences of IgE/CD23-mediated antigen presentation in allergy. *Immunol Today* 1995; **16:** 380–383.
22. Sanchez-Guerrero I, Albadejo MD, Garcia-Alonso AM, Muro M, Hernandez J, Alvarez MR. Soluble CD23 (sCD23) serum levels and lymphocyte subpopulations in peripheral blood in rhinitis and extrinsic and intrinsic asthma. *Allergy* 1994; **49:** 587–592.

ACKNOWLEDGEMENTS. This work was supported by a grant from Ministero dell'Università e della Ricerca Scientifica e Tecnologica (60%) to Dr Gabriele Di Lorenzo.

**Received 25 October 1995; accepted 6 December 1995**
